# The rationale and design of the antihypertensives and vascular, endothelial, and cognitive function (AVEC) trial in elderly hypertensives with early cognitive impairment: Role of the renin angiotensin system inhibition

**DOI:** 10.1186/1471-2318-9-48

**Published:** 2009-11-18

**Authors:** Ihab Hajjar, Meaghan Hart, William Milberg, Vera Novak, Lewis Lipsitz

**Affiliations:** 1 Harvard Medical School, Boston, MA, USA; 2 Institue for Aging Research, Hebrew SeniorLife, Boston, MA, USA; 3 Division of Gerontology, Beth Israel Deaconess Medical Centre, Boston, MA, USA; 4 GRECC VA Boston Healthcare, Boston, MA, USA

## Abstract

**Background:**

Prior evidence suggests that the renin angiotensin system and antihypertensives that inhibit this system play a role in cognitive, central vascular, and endothelial function. Our objective is to conduct a double-blind randomized controlled clinical trial, the antihypertensives and vascular, endothelial, and cognitive function (AVEC), to compare 1 year treatment of 3 antihypertensives (lisinopril, candesartan, or hydrochlorothiazide) in their effect on memory and executive function, cerebral blood flow, and central endothelial function of seniors with hypertension and early objective evidence of executive or memory impairments.

**Methods/Design:**

The overall experimental design of the AVEC trial is a 3-arm double blind randomized controlled clinical trial. A total of 100 community eligible individuals (60 years or older) with hypertension and early cognitive impairment are being recruited from the greater Boston area and randomized to lisinopril, candesartan, or hydrochlorothiazide ("active control") for 12 months. The goal of the intervention is to achieve blood pressure control defined as SBP < 140 mm Hg and DBP < 90 mm Hg. Additional antihypertensives are added to achieve this goal if needed. Eligible participants are those with hypertension, defined as a blood pressure 140/90 mm Hg or greater, early cognitive impairment without dementia defined (10 or less out of 15 on the executive clock draw test or 1 standard deviation below the mean on the immediate memory subtest of the repeatable battery for the assessment of neuropsychological status and Mini-Mental-Status-exam >20 and without clinical diagnosis of dementia or Alzheimer's disease). Individuals who are currently receiving antihypertensives are eligible to participate if the participants and the primary care providers are willing to taper their antihypertensives. Participants undergo cognitive assessment, measurements of cerebral blood flow using Transcranial Doppler, and central endothelial function by measuring changes in cerebral blood flow in response to changes in end tidal carbon dioxide at baseline (off antihypertensives), 6, and 12 months. Our outcomes are change in cognitive function score (executive and memory), cerebral blood flow, and carbon dioxide cerebral vasoreactivity.

**Discussion:**

The AVEC trial is the first study to explore impact of antihypertensives in those who are showing early evidence of cognitive difficulties that did not reach the threshold of dementia. Success of this trial will offer new therapeutic application of antihypertensives that inhibit the renin angiotensin system and new insights in the role of this system in aging.

**Trial Registration:**

Clinicaltrials.gov NCT00605072

## Background

In addition to its role in developing cardiovascular disease and stroke, hypertension is also a risk factor for cognitive impairment [1-5] Although blood pressure tends to decline around the time of onset of clinical cognitive impairment[3], hypertension leads to accelerated decline in those with cognitive impairment or dementia[6,7] Of all the cognitive domains, executive function is more vulnerable to the effects of hypertension [8-10] Executive function is defined as the set of cognitive skills that are responsible for the planning, initiation, sequencing, and monitoring of complex goal-directed behavior[11] Seniors suffering from executive dysfunction have significant impairment in following medical advise [12] and are more likely to develop disability[13,14] It is estimated that close to 30% of the elderly population have executive function abnormalities and are generally undetected[15,16] No prior research has evaluated the effect of hypertension treatment on executive function or specifically enrolled those with executive dysfunction.

The process by which hypertension can affect cognitive and executive function is not clear. It is likely to be, in part, related to the cerebral blood flow (CBF) regulatory system. This is supported by the evidence that lower CBF measured by Transcranial Doppler (TCD) is associated with progressive decline in cognitive function[17] For example, in patients with amnestic mild cognitive impairment, lower CBF was associated with a higher risk for converting to dementia[18] Further, abnormal cerebrovascular reactivity was associated with worsening cognitive decline in patients with Alzheimer's disease[19] Hypertension is associated with a decrease in CBF over and above any effect of age [20-23] Hypertension also impairs neurovascular coupling[24] and vasoreactivity to CO_2_, a measure of brain endothelial function[25] Taken together, this provides a rational for investigating the role of CBF regulation in the relation between hypertension and cognitive function.

From a neuro-humoral standpoint, multiple systems may be involved in the relation between hypertension and cognitive function. However, a paucity of evidence point to the renin angiotensin system (RAS). Anatomically, angiotensin II (Ang II) and its receptors are located in neurons inside the blood brain barrier and in the cerebrovascular endothelial cells and circumventricular organs[26] Functionally, Ang II has been linked with cognitive function in animal models[27] In addition, Ang II also decreases cerebral blood flow[28] and impairs neurovascular coupling[29] in hypertensive patients.

Ang II impairs endothelial function, which has been linked to poor cognitive function and early Alzheimer's disease [30-33] The role of the endothelium in cognitive function, aging and hypertension has gained much attention recently [33-38] Central endothelial function can be assessed indirectly by the response of CBF to changes in end-tidal CO_2_[39] Ang II plays a critical pathophysiological role in impairing endothelial function, especially in those with hypertension[30-32,40] Therefore we are suggesting that hypertension is associated with RAS activation and endothelial function impairment that in turn are associated with abnormal CBF regulation and cognitive and executive function impairments.

Multiple observational and experimental studies, although not universally consistent, have shown that use of antihypertensives may provide cognitive protection in the elderly population[41] However, it is not known if they provide this effect solely by lowering blood pressure or via an additional class specific effect[42] Based on our suggested pathway from hypertension to cognitive impairment, we are hypothesizing that antihypertensives that inhibit the activity of RAS lower this risk beyond just lowering blood pressure. They also restore endothelial function and cerebral blood flow regulation leading to further cognitive protection.

Drugs that inhibit RAS include angiotensin converting enzyme inhibitors (ACEI) and angiotensin receptor blockers (ARB). ACEI block ACE and decrease Ang II production, whereas ARB block the angiotensin receptor type 1 but not type 2[43,44] ACEI and ARB may be protective against cognitive and physical impairment in hypertensives [45-50] In an observational study of 1,220 Italian individuals with heart failure, treatment with ACEI was associated with improved cognitive performance[51] The Perindopril Protection Against Recurrent Stroke Study indicated that perindopril (ACEI) reduced the risk of incident cognitive impairment in those with a previous history of stroke[46] In the Cognition and Prognosis in the Elderly trial, treatment with candesartan (ARB) was associated with a lower rate of cognitive decline only in those with Mini-Mental Status-Examination Scores between 24 and 28 compared to placebo, but not the whole sample[52] Other antihypertensives such as diuretics and calcium channel blockers may increase RAS activity through increasing renin levels[53,54] Diuretics, which produce similar lowering blood pressure effect to ACEI and ARB, activate RAS. Studies suggest that diuretics do not demonstrate a cognitive protective effect [55].

In addition to their effect on cognitive function, animal and human studies have suggested that both ACEI and ARB are associated with improved cerebral hemodynamics[56,57] In spontaneously hypertensive rats, treatment with ARB was associated with improved cerebral autoregulation[58], reduction in CBF decline after middle cerebral artery occlusions,[59] and normalization of the production of nitric oxide[60]. Treatment with candesartan or captopril (ACEI) was also associated with a smaller infract size after middle cerebral artery occlusion[59] In addition, ARB has a specific anti-aging effect on the cardiovascular system. For example, losartan, but not diuretic-based antihypertensive treatments, reduced heart to body weight ratio in old but not young Wister-Kyoto rats[61] In humans, studies on the effect of ACEI or ARB on cerebral hemodynamics are scarce. Work by our coauthor, Dr. Lipsitz, demonstrated that ACEI, but not other antihypertensives, improve CBF and cerebral vasoreactivity in cognitively intact hypertensive older adults[62,63] ARB have also been found to improve cerebral autoregulation in hypertensive patients with strokes[64] and diabetes[65] Preliminary studies suggest that drugs that inhibit RAS improve endothelial function[66] For example, in hypertensive patients treated with a beta-blocker, switching to an ARB resulted in improved endothelial function, measured by flow mediated dilatation[67]

Of the two classes, recent studies have shown that ARB may be superior to ACEI or diuretics in providing neuro-protection and improving vascular function[45,64,68] Both type 1 and 2 angiotensin receptors are present in the brain and have opposing effects: type-1 leads to vasoconstriction, whereas type 2 receptor leads to vasodilatation, neuronal differentiation, apoptosis and axonal regeneration[69] ARB, but not ACEI inhibit the type-1 receptors leaving the type-2 receptors active. This may translate to greater cerebral dilatation and a superior protective effect. In fact, preliminary evidence suggests that ARB are superior to other antihypertensives in their ability to reduce the risk of stroke[70] They also provide greater anti-atherosclerotic effect; measured by intima-media thickness, compared to ACEI in elderly hypertensive individuals[71] Therefore, we hypothesize that ARB are superior to ACEI in their cognitive and endothelial function effect measured by TCD at the middle cerebral artery, which in turn is superior to HCTZ, a drug that activates RAS.

Our objective is to conduct a double-blind randomized controlled clinical trial, the antihypertensives and vascular, endothelial, and cognitive function (AVEC), to compare 1 year treatment of 3 antihypertensives (lisinopril, candesartan, or hydrochlorothiazide (HCTZ)) in their effect on memory and executive function, cerebral blood flow, and central endothelial function of seniors with hypertension and early objective evidence of executive or memory decline.

## Methods/Design

### Experimental design and participants

**Figure 1:**
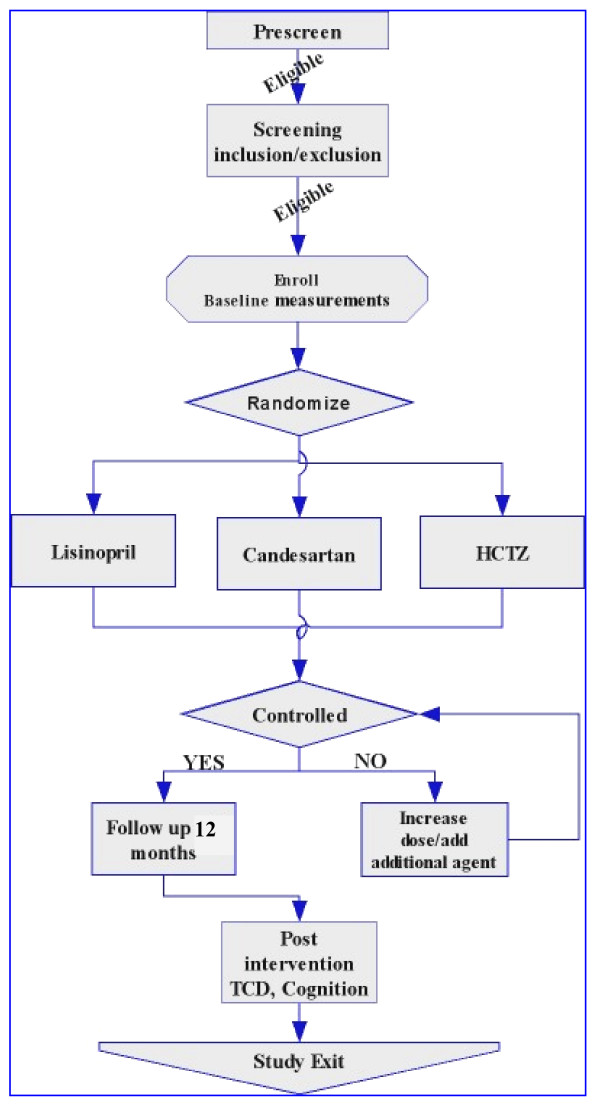
**Study flow of the AVEC trial from the screening tothe study exit**.

The overall experimental design of the AVEC trial is a 3-arm double blind randomized controlled clinical trial. A total of 100 community eligible individuals with hypertension and early cognitive impairment are being recruited from the greater Boston area. Evaluated at the cardiovascular research laboratory at the Institute for Aging Research, and then randomized to candesartan, lisinopril, or HCTZ ("active control") for 12 months. Currently recruitment efforts include various community activities, faith-based community events, health fairs, advertisements, and mail out announcements. Figure 1 describes the flow from screening to study exit. The Institutional Review Board at the Institute for Aging Research at Hebrew Rehabilitation Center for Aged approved the study and all participants provide written informed consent.

### Inclusion Criteria include

(1) 60 years or older; (2) hypertension: defined as a systolic blood pressure (SBP) of 140 mm Hg or greater or diastolic blood pressure (DBP) 90 mm Hg or greater or receiving antihypertensive medications; (3) early cognitive impairment without dementia defined as executive impairment (measured by the executive clock draw test (CLOX)) [72] or early memory impairment (measured by the repeatable battery for the assessment of neuropsychological status) [73]. Both of these tests are brief (total of 15 minutes), have been validated in elderly populations and have standardized norms[72,74]

### Exclusion criteria include

(1) Intolerance to 2 of the study medications; (2) SBP >200/DBP >110 mm Hg if not on treatment or SBP > 180/DBP > 100 mm Hg if on antihypertensive therapy; (3) Mini-Mental-Status-exam (MMSE)<20 or a clinical diagnosis of dementia or Alzheimer's disease; (4) elevated serum creatinine or serum potassium at baseline; (5) receiving >2 antihypertensives; (6) Comorbid illnesses (congestive heart failure, diabetes mellitus stroke); and (7) inability to perform the study procedures.

Individuals who are currently receiving 1 or 2 antihypertensives are eligible to participate if the participant and the primary care provider are willing to taper their antihypertensives. The medication is tapered while carefully monitoring blood pressure using a standard protocol in our laboratory [63].

### The intervention

The intervention is treatment with lisinopril, candesartan, or HCTZ. The goal of the intervention is to achieve blood pressure control defined as SBP < 140 mm Hg and DBP < 90 mm Hg. The starting dose is 10 mg increased to 20 mg then 40 mg of lisinopril; 8 mg increased to 16 mg then 32 mg of candesartan; and 12.5 mg increased to 25 mg of HCTZ. Titration occurs every 2 weeks until blood pressure is <140/90 mm Hg. If blood pressure is still not controlled, long acting nifedipine is added at 30 mg increased to 60 mg and 90 mg in 2 week increments. If still not controlled, a beta-blocker is added at 12.5 mg increased to 25 mg and 50 mg of long-acting metoprolol.

### Randomization and blinding

The randomization is performed using computer generated random numbers leading to a random allocation sequence. This random allocation is performed at the central pharmacy at Hebrew SeniorLife. Both study personnel and participants are blinded to the group assignment.

### Study phases

The following are the study phases:

(i) Screen: This includes: (1) informed consent, (2) 2 seated blood pressure measurements separated by 5 minutes, (3) executive function and memory assessments, and (4) inclusion/exclusion criteria assessment. Eligible participants are scheduled for the Baseline visit. If they are receiving antihypertensives, they are also given the automated blood pressure measurement machine. Instructions on using the machine, tapering the antihypertensives, frequency of measurements, a diary, and appropriate phone numbers are provided.

(ii) Baseline evaluation and randomization: during this visit, participants undergo blood pressure measurements, cognitive assessments, physical measures, and TCD procedures. Randomization to one of the 3 antihypertensive medications occurs after the baseline visit.

(iii) Follow up: Participants are then seen every 2 weeks until blood pressure control (<140/90 mm Hg) is achieved. During these visits, blood pressure measurements, medication adjustments and assessments of potential adverse events are completed. Participants are then followed for 12 months after achieving target blood pressure (<140/90 mm Hg) and evaluated at 1, 3, 6, and 12 months. At each evaluation, blood pressure, adverse events, pill count (to assess compliance), use of other medications is assessed, and blood samples are drawn as outlined in Table 1. A re-evaluation including cognitive assessment, physical measures, TCD, and medical examination are performed at 6 and 12 months.

**Table 1:** Study procedures at various stages of the AVEC trial

*Visit*	*Screen*	*Enrollment*	*Baseline*	Titrate*	*Follow-up*		
Months	0	2-4 weeks	1	2-3	3	6	12

Informed Consent/Eligibility	X	X				X	

Medication Inventory		X	X	X	X	X	X

Weight/Height			X			X	X

Blood pressure (2 every 5 min)	X	X	X	X	X	X	X

Psychological Assessment	X		X			X	X

Cerebrovascular Assessment			X			X	X

Biochemical Measures		X		X	X	X	X

Renin/Aldosterone			X			X	X

Adverse Events Screening				X	X	X	X

If a titration of medication needed baseline will be performed after titration and before randomization

### Experimental Procedures and measures

(i) Health Interview and exam: Demographics, social history and habits, family history, medication inventory[75], physical activity using the Physical Activity Scale for the Elderly[76] and Instrumental Activities of Daily Living (IADL) [77] data will be collected. Blood pressure is measured using standardized procedures according to the American Heart Association guidelines[78,79]: the participant is in the sitting position, rested for 5 minutes, no caffeine or smoking 2 hours prior to measurement, appropriate cuff size (covering 60% of upper arm length and 80% of arm circumference), correct cuff placement (1-2 inches above brachial pulse on bare arm), and use of the bell of the stethoscope or an automatic calibrated blood pressure machine. The systolic blood pressure is defined as the pressure corresponding to the first korotkoff sound (K1) and the diastolic as the pressure corresponding to the last korotkoff sound (K5). Blood pressure is measured in both arms and recorded. The arm with the higher blood pressure is used throughout the study. Two blood pressure readings are performed at each visit and averaged per visit. The physical exam includes gait speed (time to walk 4 meters conducted twice), height, and weight measurements.

(ii) Neuropsychological assessment: The battery of tests we chose for both screening and for the outcome measures was selected with the following considerations: brevity, validation in a similar elderly population, and sensitivity to detect impairment change over the study period.

The screening tests include: the executive clock draw test part 1 (CLOX1) and the Repeatable Battery for the Assessment of Neuropsychological Status (RBANS) immediate memory domain. We selected the CLOX1 as a screening tool to detect those with executive function abnormalities. The subject draws a clock in response to the examiner's request. CLOX1 is sensitive to detect executive function[72] It has high internal consistency (Cronbach's alpha = 0.82) and high between-rater reliability (r = 0.94, p < 0.001)[72] A score 10 or less on CLOX1 is required for eligibility[72] The RBANS immediate memory domain is a brief test to assess memory [74]. Participants are asked to repeat a list of 10 words and to repeat a short story. The reliability coefficient is 88% for the immediate memory domain. Those who score 1 standard deviation or below the age-education-specific standardized mean on this test will be eligible for the study. The Mini-Mental-Status-Examination (MMSE) is used to exclude participants with more advanced cognitive impairment. We selected this test since those who score 20 or less are more likely to have advanced cognitive impairment and dementia [80]. The inter-rater reliability of MMSE is 0.83[81]

The study outcome cognitive tests include Trail Making test (A and B), Hopkins Verbal Learning Test - Revised (HVLT-R), and the Digit Span Test. The Trail Making test requires the connection of sequentially numbered circles (A), and the connection of circles marked by numbers and letters in an alternating sequence (B). This test is considered a benchmark of executive function. We *selected *this test because it is sensitive to detect frontal lobe pathology in patients with increased cerebrovascular and cardiovascular risk such as those with hypertension[8,10] The HVLT-R is a 12-item list learning test in which individuals are presented three learning and recall trials followed by a delayed recall and 24 item recognition test. The HVLT-R has been identified as an ideal memory measure for elderly patients, and appropriate reliability and validity have been shown in older individuals[82] We selected this test because it is brief and allows us to identify abnormalities in immediate vs. delayed recognition. The Digit Span Test is a brief task that assesses immediate memory/attention. It is administered using the standard format[83] It consists of a series of digits of increasing length, some of which are recited as presented, and some of which are to be recited in the reversed order[84] We *selected *this test since it can detect abnormalities with encoding.

(iii) Cerebral Blood Flow and its regulation: CBF autoregulation and cerebral vasoreactivity are assessed by TCD using standard procedures described previously[85] Blood flow velocity, and hence CBF, is measured at the middle cerebral artery, which has a high correlation coefficient (0.995) with invasive blood flow measurements and high reliability in elderly subjects [86]. Beat to beat heart rate and blood pressure are also measured by the Finapress system and EKG[87] The Sit-to-Stand is used to assess autoregulation by measuring continuous CBF, blood pressure and heart rate in response to postural changes in sitting and standing positions. Vasoreactivity is measured by asking participants to deeply breath air with 8% CO_2 _[88] followed by hyperventilation. CBF, blood pressure, and heart rate are continuously measured during hyper and hypocapnea. The change in CBF relative to the change in mean arterial pressure between sitting and standing is used to measure autoregulation. The slope of the relation between cerebrovascular conductance (mean CBF/mean arterial blood pressure) for each R-R interval and end-tidal CO_2 _is used to assess CO_2 _vasoreactivity [88].

(iv) Biochemical measurement is done to monitor for adverse events: hyperkalemia and renal failure. RAS activity is assessed using plasma renin activity and aldosterone levels[89] Plasma aldosterone is measured by a radioimmunometric assay (RIA). The Coat-A-Count procedure is a solid phase radioimmunoassay, based on aldosterone specific antibody immobilized to the wall of the polypropylene tube. 125I-labelled aldosterone competes for a fixed time with aldosterone in the patient sample for antibody sites. The sensitivity of this method is 16 pg/mL and the precision is 4-10%[90]. Plasma renin activity is measured by competitive binding radioimmunoassay (RIA) using the GammaCoat plasma renin activity RIA kit. The determination involves an initial incubation of plasma to generate angiotensin I, followed by quantification of angiotensin I by RIA. This technique is highly sensitive to low levels of 0.01 ng/mL/hour and is precise to activity that is less than 10%[91] In addition, a blood sample is drawn for storage and DNA extraction for future genetic studies.

(v) Adverse events (AE)*: *We are screening for potential adverse events through self-report by the participant or the next of kin. These include: dizziness, vertigo, light-headedness, weakness, swollen lip or tongue or both, hospitalizations, cough, falls, angioedema, renal insufficiency, and hyperkalemia with ACEI and ARB. Hypokalemia may occur with HCTZ (potassium will be supplemented). The incidence of cough with ACEI varies but 5% is the most commonly reported rate [92]. Angioedema is extremely rare and occurs in about 0.68% of patients treated with ACEI [93]. Screening for adverse events occurs monthly by phone and during each visit where measurements of serum potassium and creatinine are performed. Emergency contact is also provided in case adverse events occur in-between visits or communications with the study personnel.

### Statistical methods

**Figure 2:**
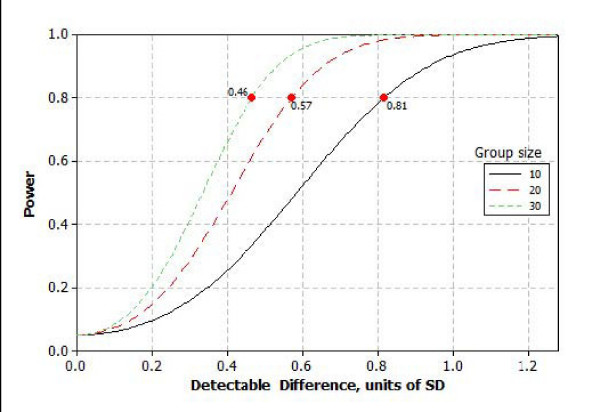
**Power analysis for the minimum detectable difference between the 3 groups in the change in outcome from baseline to 12 months of the AVEC trial**. (assuming alpha = 0.05 and power = 80%)

All analyses will follow the *intention-to-treat *principle. We will use Mixed Models since we have correlated repeat observations and this procedure is not significantly affected by missing data[94,95] We will compare the change in the cognitive or CBF outcomes from baseline to 12 months between the three groups. The *primary independent variable *is group assignment (candesartan, lisinopril, or hydrochlorothiazide). A sample size of 100 will allow us to successfully complete at least 90 (10% drop out, 30 per group) participants. A 30 per group (90 total sample size) allows us to detect at least between group differences in the change (from baseline to 12 months) of 0.46 standard deviation units (α = 0.05; power 80%). Figure 2 provides results of power analysis for the detectable difference, sample size and power. Analysis for the safety of the intervention will be conducted by comparing the number of adverse events (cough, angioedema, leukopinea, increase in serum creatinine by more than 1 mg/dl, hyperkalemia (> = 5.5 meq/dl), hypotension (SBP < 100), dizziness, falls and hospitalization) and the number of participants who withdrew from the study in the three groups using Analysis of Variance (ANOVA).

## Discussion

Aging of the population will lead to increase prevalence of hypertension and cognitive difficulties. To date, there are no available data to provide guidelines for treatment of hypertension in those with cognitive impairment. The AVEC trial is the first study to explore impact of antihypertensives in those who are showing early evidence of cognitive difficulties that have not reached the threshold of dementia. Observational studies have been conflicting and some suggest that treatment of hypertension may have a negative effect on cognitive function [96-98]. Therefore, a carefully performed clinical trial that selects the appropriate population to study is critical to answer the question about the impact of antihypertensives on cognitive function.

The role of the RAS in aging is important because of the available therapeutic intervention for this system. Recently, RAS is a critical system in vascular biology and recently in neuroscience. Identifying an impact of ACEI or ARB on cognitive or cerebrovascular function will provide additional mechanistic and therapeutic evidence of the role of RAS in aging.

## Competing interests

The authors declare that they have no competing interests.

## Authors' contributions

IH, LL and WM were involved in developing the basic idea for the study. VN was further involved in developing the idea and the transcranial Doppler and antihypertensive taper protocols. IH and MH are responsible for the data collection. All authors contributed to the final manuscript by reading and correcting draft versions.

## Pre-publication history

The pre-publication history for this paper can be accessed here:

http://www.biomedcentral.com/1471-2318/9/48/prepub

## References

[B1] Kilander L Nyman H Boberg M Hansson L Lithell H Hypertension is related to cognitive impairment: a 20-year follow-up of 999 men Hypertension 1998 31 780 786 9495261 10.1161/01.hyp.31.3.780

[B2] Paran E Anson O Reuveni H Blood pressure and cognitive functioning among independent elderly Am J Hypertens 2003 16 818 826 10.1016/S0895-7061(03)01005-7 14553960

[B3] Launer LJ Masaki K Petrovitch H Foley D Havlik RJ The association between midlife blood pressure levels and late-life cognitive function. The Honolulu-Asia Aging Study Jama 1995 274 1846 1851 10.1001/jama.274.23.1846 7500533

[B4] Knopman D Boland LL Mosley T Howard G Liao D Szklo M McGovern P Folsom AR Cardiovascular risk factors and cognitive decline in middle-aged adults Neurology 2001 56 42 48 11148234 10.1212/wnl.56.1.42

[B5] Reinprecht F Elmstahl S Janzon L Andre-Petersson L Hypertension and changes of cognitive function in 81-year-old men: a 13-year follow-up of the population study "Men born in 1914", Sweden J Hypertens 2003 21 57 66 10.1097/00004872-200301000-00014 12544436

[B6] Bellew KM Pigeon JG Stang PE Fleischman W Gardner RM Baker WW Hypertension and the rate of cognitive decline in patients with dementia of the Alzheimer type Alzheimer Dis Assoc Disord 2004 18 208 213 15592132

[B7] Goldstein FC Ashley AV Freedman LJ Penix L Lah JJ Hanfelt J Levey AI Hypertension and cognitive performance in African Americans with Alzheimer disease Neurology 2005 64 899 901 10.1212/01.WNL.0000164712.24389.BB 15753433

[B8] Pugh KG Kiely DK Milberg WP Lipsitz LA Selective impairment of frontal-executive cognitive function in african americans with cardiovascular risk factors J Am Geriatr Soc 2003 51 1439 1444 10.1046/j.1532-5415.2003.51463.x 14511165 PMC4415529

[B9] Saxby BK Harrington F McKeith IG Wesnes K Ford GA Effects of hypertension on attention, memory, and executive function in older adults Health Psychol 2003 22 587 591 10.1037/0278-6133.22.6.587 14640855

[B10] Kuo HK Sorond F Iloputaife I Gagnon M Milberg W Lipsitz LA Effect of blood pressure on cognitive functions in elderly persons J Gerontol A Biol Sci Med Sci 2004 59 1191 1194 15602074 10.1093/gerona/59.11.1191 PMC4418553

[B11] Royall DR Lauterbach EC Cummings JL Reeve A Rummans TA Kaufer DI LaFrance WC Jr Coffey CE Executive Control Function: A Review of Its Promise and Challenges for Clinical Research. A Report From the Committee on Research of the American Neuropsychiatric Association J Neuropsychiatry Clin Neurosci 2002 14 377 405 12426407 10.1176/jnp.14.4.377

[B12] Royall DR Cordes J Polk M Executive control and the comprehension of medical information by elderly retirees Exp Aging Res 1997 23 301 313 10.1080/03610739708254033 9352289

[B13] Cahn-Weiner DA Malloy PF Boyle PA Marran M Salloway S Prediction of functional status from neuropsychological tests in community-dwelling elderly individuals Clin Neuropsychol 2000 14 187 195 10916193 10.1076/1385-4046(200005)14:2;1-Z;FT187

[B14] Royall DR Palmer R Chiodo LK Polk MJ Declining executive control in normal aging predicts change in functional status: the Freedom House Study J Am Geriatr Soc 2004 52 346 352 10.1111/j.1532-5415.2004.52104.x 14962147

[B15] Grigsby J Kaye K Shetterly SM Baxter J Morgenstern NE Hamman RF Prevalence of disorders of executive cognitive functioning among the elderly: findings from the San Luis Valley Health and Aging Study Neuroepidemiology 2002 21 213 220 10.1159/000065638 12207148

[B16] Royall DR Espino DV Polk MJ Palmer RF Markides KS Prevalence and patterns of executive impairment in community dwelling Mexican Americans: results from the Hispanic EPESE Study Int J Geriatr Psychiatry 2004 19 926 934 10.1002/gps.1185 15449370

[B17] Ruitenberg A den Heijer T Bakker SL van Swieten JC Koudstaal PJ Hofman A Breteler MM Cerebral hypoperfusion and clinical onset of dementia: the Rotterdam Study Ann Neurol 2005 57 789 794 10.1002/ana.20493 15929050

[B18] Maalikjy Akkawi N Borroni B Agosti C Magoni M Broli M Pezzini A Padovani A Volume cerebral blood flow reduction in pre-clinical stage of Alzheimer disease: evidence from an ultrasonographic study J Neurol 2005 252 559 563 10.1007/s00415-005-0689-z 15726249

[B19] Silvestrini M Pasqualetti P Baruffaldi R Bartolini M Handouk Y Matteis M Moffa F Provinciali L Vernieri F Cerebrovascular reactivity and cognitive decline in patients with Alzheimer disease Stroke 2006 37 1010 1015 10.1161/01.STR.0000206439.62025.97 16497984

[B20] Ameriso SF Paganini-Hill A Meiselman HJ Fisher M Correlates of middle cerebral artery blood velocity in the elderly Stroke 1990 21 1579 1583 2237952 10.1161/01.str.21.11.1579

[B21] Murphy DG DeCarli C McIntosh AR Daly E Mentis MJ Pietrini P Szczepanik J Schapiro MB Grady CL Horwitz B Rapoport SI Sex differences in human brain morphometry and metabolism: an in vivo quantitative magnetic resonance imaging and positron emission tomography study on the effect of aging Arch Gen Psychiatry 1996 53 585 594 8660125 10.1001/archpsyc.1996.01830070031007

[B22] Krejza J Mariak Z Walecki J Szydlik P Lewko J Ustymowicz A Transcranial color Doppler sonography of basal cerebral arteries in 182 healthy subjects: age and sex variability and normal reference values for blood flow parameters AJR Am J Roentgenol 1999 172 213 218 9888770 10.2214/ajr.172.1.9888770

[B23] Cavestri R Radice L Ferrarini F Longhini M Longhini E Influence of erythrocyte aggregability and plasma fibrinogen concentration on CBF with aging Acta Neurol Scand 1992 85 292 298 10.1111/j.1600-0447.1992.tb01472.x 1585800

[B24] Kazama K Wang G Frys K Anrather J Iadecola C Angiotensin II attenuates functional hyperemia in the mouse somatosensory cortex Am J Physiol Heart Circ Physiol 2003 285 H1890 1899 12907423 10.1152/ajpheart.00464.2003

[B25] Serrador JM Sorond FA Vyas M Gagnon M Iloputaife ID Lipsitz LA Cerebral pressure-flow relations in hypertensive elderly humans: transfer gain in different frequency domains J Appl Physiol 2005 98 151 159 10.1152/japplphysiol.00471.2004 15361517

[B26] Ando H Zhou J Macova M Imboden H Saavedra JM Angiotensin II AT1 receptor blockade reverses pathological hypertrophy and inflammation in brain microvessels of spontaneously hypertensive rats Stroke 2004 35 1726 1731 10.1161/01.STR.0000129788.26346.18 15143297

[B27] Gard PR The role of angiotensin II in cognition and behaviour Eur J Pharmacol 2002 438 1 14 10.1016/S0014-2999(02)01283-9 11906704

[B28] Saavedra JM Nishimura Y Angiotensin and cerebral blood flow Cell Mol Neurobiol 1999 19 553 573 10.1023/A:1006995016403 10384255 PMC11545525

[B29] Kazama K Anrather J Zhou P Girouard H Frys K Milner TA Iadecola C Angiotensin II impairs neurovascular coupling in neocortex through NADPH oxidase-derived radicals Circ Res 2004 95 1019 1026 10.1161/01.RES.0000148637.85595.c5 15499027

[B30] Haberl RL Decker PJ Einhaupl KM Angiotensin degradation products mediate endothelium-dependent dilation of rabbit brain arterioles Circ Res 1991 68 1621 1627 2036715 10.1161/01.res.68.6.1621

[B31] Dal-Ros S Bronner C Schott C Kane MO Chataigneau M Schini-Kerth VB Chataigneau T Angiotensin II-induced hypertension is associated with a selective inhibition of endothelium-derived hyperpolarizing factor-mediated responses in the rat mesenteric artery J Pharmacol Exp Ther 2009 328 478 486 10.1124/jpet.108.145326 18984652

[B32] Dimitropoulou C Chatterjee A McCloud L Yetik-Anacak G Catravas JD Angiotensin, bradykinin and the endothelium Handb Exp Pharmacol 2006 255 294 full_text 16999222 10.1007/3-540-32967-6_8

[B33] Khalil Z LoGiudice D Khodr B Maruff P Masters C Impaired peripheral endothelial microvascular responsiveness in Alzheimer's disease J Alzheimers Dis 2007 11 25 32 17361032 10.3233/jad-2007-11106

[B34] Ghiadoni L Virdis A Aging and hypertension: what about the endothelium? J Hypertens 2006 24 1243 1244 10.1097/01.hjh.0000234100.93120.c5 16794469

[B35] Versari D Daghini E Virdis A Ghiadoni L Taddei S The ageing endothelium, cardiovascular risk and disease in man Exp Physiol 2009 94 317 321 10.1113/expphysiol.2008.043356 18805864

[B36] Taddei S Virdis A Ghiadoni L Versari D Salvetti A Endothelium, aging, and hypertension Curr Hypertens Rep 2006 8 84 89 10.1007/s11906-006-0045-4 16600164

[B37] d'Alessio P Aging and the endothelium Exp Gerontol 2004 39 165 171 10.1016/j.exger.2003.10.025 15038389

[B38] Moser DJ Miller IN Hoth KF Correia M Arndt S Haynes WG Vascular smooth muscle function is associated with initiation and processing speed in patients with atherosclerotic vascular disease J Int Neuropsychol Soc 2008 14 535 541 10.1017/S1355617708080697 18577282 PMC3597121

[B39] Ainslie PN Murrell C Peebles K Swart M Skinner MA Williams MJ Taylor RD Early morning impairment in cerebral autoregulation and cerebrovascular CO2 reactivity in healthy humans: relation to endothelial function Exp Physiol 2007 92 769 777 10.1113/expphysiol.2006.036814 17384117

[B40] Ai D Fu Y Guo D Tanaka H Wang N Tang C Hammock BD Shyy JY Zhu Y Angiotensin II up-regulates soluble epoxide hydrolase in vascular endothelium in vitro and in vivo Proc Natl Acad Sci USA 2007 104 9018 9023 10.1073/pnas.0703229104 17495027 PMC1885620

[B41] Murray MD Lane KA Gao S Evans RM Unverzagt FW Hall KS Hendrie H Preservation of cognitive function with antihypertensive medications: a longitudinal analysis of a community-based sample of African Americans Arch Intern Med 2002 162 2090 2096 10.1001/archinte.162.18.2090 12374517

[B42] Manolio TA Olson J Longstreth WT Hypertension and cognitive function: pathophysiologic effects of hypertension on the brain Curr Hypertens Rep 2003 5 255 261 10.1007/s11906-003-0029-6 12724059

[B43] Roig E Perez-Villa F Morales M Jimenez W Orus J Heras M Sanz G Clinical implications of increased plasma angiotensin II despite ACE inhibitor therapy in patients with congestive heart failure Eur Heart J 2000 21 53 57 10.1053/euhj.1999.1740 10610744

[B44] Padmanabhan N Jardine AG McGrath JC Connell JM Angiotensin-converting enzyme-independent contraction to angiotensin I in human resistance arteries Circulation 1999 99 2914 2920 10359736 10.1161/01.cir.99.22.2914

[B45] Tedesco MA Ratti G Mennella S Manzo G Grieco M Rainone AC Iarussi D Iacono A Comparison of losartan and hydrochlorothiazide on cognitive function and quality of life in hypertensive patients Am J Hypertens 1999 12 1130 1134 10.1016/S0895-7061(99)00156-9 10604491

[B46] Tzourio C Anderson C Chapman N Woodward M Neal B MacMahon S Chalmers J Effects of blood pressure lowering with perindopril and indapamide therapy on dementia and cognitive decline in patients with cerebrovascular disease Arch Intern Med 2003 163 1069 1075 10.1001/archinte.163.9.1069 12742805

[B47] Cummings JL Cole G Alzheimer disease Jama 2002 287 2335 2338 10.1001/jama.287.18.2335 11988038

[B48] Benedict RH Brandt J Limitation of the Mini-Mental State Examination for the detection of amnesia J Geriatr Psychiatry Neurol 1992 5 233 237 1418369 10.1177/002383099200500409

[B49] Pogosova GV Zhidko NI Ivanishina NS Gudkova OA Avakian GN [Ramipril in elderly patients with mild and moderate hypertension. Clinical efficacy, effect on cerebral blood flow and intellectual functioning] Kardiologiia 2003 43 42 47 12891310

[B50] Braszko JJ Karwowska-Polecka W Halicka D Gard PR Captopril and enalapril improve cognition and depressed mood in hypertensive patients J Basic Clin Physiol Pharmacol 2003 14 323 343 15198305 10.1515/jbcpp.2003.14.4.323

[B51] Zuccala G Onder G Marzetti E Monaco MR Cesari M Cocchi A Carbonin P Bernabei R Use of angiotensin-converting enzyme inhibitors and variations in cognitive performance among patients with heart failure Eur Heart J 2005 26 226 233 10.1093/eurheartj/ehi058 15618043

[B52] Skoog I Lithell H Hansson L Elmfeldt D Hofman A Olofsson B Trenkwalder P Zanchetti A Effect of baseline cognitive function and antihypertensive treatment on cognitive and cardiovascular outcomes: Study on COgnition and Prognosis in the Elderly (SCOPE) Am J Hypertens 2005 18 1052 1059 10.1016/j.amjhyper.2005.02.013 16109319

[B53] Wagner C Kramer BK Hinder M Kieninger M Kurtz A T-type and L-type calcium channel blockers exert opposite effects on renin secretion and renin gene expression in conscious rats Br J Pharmacol 1998 124 579 585 10.1038/sj.bjp.0701861 9647484 PMC1565416

[B54] Nascimento L Ayala JM Baquero RA Martinez-Maldonado M Renin release by diuretics J Pharmacol Exp Ther 1979 208 522 526 430369

[B55] Di Bari M Pahor M Franse LV Shorr RI Wan JY Ferrucci L Somes GW Applegate WB Dementia and disability outcomes in large hypertension trials: lessons learned from the systolic hypertension in the elderly program (SHEP) trial Am J Epidemiol 2001 153 72 78 10.1093/aje/153.1.72 11159149

[B56] Sadoshima S Nagao T Ibayashi S Fujishima M Inhibition of angiotensin-converting enzyme modulates the autoregulation of regional cerebral blood flow in hypertensive rats Hypertension 1994 23 781 785 8206577 10.1161/01.hyp.23.6.781

[B57] Dupuis F Atkinson J Liminana P Chillon JM Captopril improves cerebrovascular structure and function in old hypertensive rats Br J Pharmacol 2005 144 349 356 10.1038/sj.bjp.0706001 15655534 PMC1576005

[B58] Nishimura Y Ito T Saavedra JM Angiotensin II AT(1) blockade normalizes cerebrovascular autoregulation and reduces cerebral ischemia in spontaneously hypertensive rats Stroke 2000 31 2478 2486 11022082 10.1161/01.str.31.10.2478

[B59] Ito T Yamakawa H Bregonzio C Terron JA Falcon-Neri A Saavedra JM Protection against ischemia and improvement of cerebral blood flow in genetically hypertensive rats by chronic pretreatment with an angiotensin II AT1 antagonist Stroke 2002 33 2297 2303 10.1161/01.STR.0000027274.03779.F3 12215602

[B60] Yamakawa H Jezova M Ando H Saavedra JM Normalization of endothelial and inducible nitric oxide synthase expression in brain microvessels of spontaneously hypertensive rats by angiotensin II AT1 receptor inhibition J Cereb Blood Flow Metab 2003 23 371 380 10.1097/00004647-200303000-00012 12621312

[B61] Demirci B McKeown PP Bayraktutan U Blockade of angiotensin II provides additional benefits in hypertension- and ageing-related cardiac and vascular dysfunctions beyond its blood pressure-lowering effects J Hypertens 2005 23 2219 2227 10.1097/01.hjh.0000191906.03983.ee 16269964

[B62] Hatazawa J Shimosegawa E Osaki Y Ibaraki M Oku N Hasegawa S Nagata K Hirata Y Miura Y Long-term angiotensin-converting enzyme inhibitor perindopril therapy improves cerebral perfusion reserve in patients with previous minor stroke Stroke 2004 35 2117 2122 10.1161/01.STR.0000136034.86144.e9 15256675

[B63] Lipsitz LA Gagnon M Vyas M Iloputaife I Kiely DK Sorond F Serrador J Cheng DM Babikian V Cupples LA Antihypertensive therapy increases cerebral blood flow and carotid distensibility in hypertensive elderly subjects Hypertension 2005 45 216 221 10.1161/01.HYP.0000153094.09615.11 15655124

[B64] Moriwaki H Uno H Nagakane Y Hayashida K Miyashita K Naritomi H Losartan, an angiotensin II (AT1) receptor antagonist, preserves cerebral blood flow in hypertensive patients with a history of stroke J Hum Hypertens 2004 18 693 699 10.1038/sj.jhh.1001735 15129230

[B65] Kario K Ishikawa J Hoshide S Matsui Y Morinari M Eguchi K Ishikawa S Shimada K Diabetic brain damage in hypertension: role of renin-angiotensin system Hypertension 2005 45 887 893 10.1161/01.HYP.0000163460.07639.3f 15824198

[B66] Morimoto S Maki K Aota Y Sakuma T Iwasaka T Beneficial effects of combination therapy with angiotensin II receptor blocker and angiotensin-converting enzyme inhibitor on vascular endothelial function Hypertens Res 2008 31 1603 1610 10.1291/hypres.31.1603 18971536

[B67] Schiffrin EL Park JB Pu Q Effect of crossing over hypertensive patients from a beta-blocker to an angiotensin receptor antagonist on resistance artery structure and on endothelial function J Hypertens 2002 20 71 78 10.1097/00004872-200201000-00011 11791028

[B68] Braszko JJ AT(2) but not AT(1) receptor antagonism abolishes angiotensin II increase of the acquisition of conditioned avoidance responses in rats Behav Brain Res 2002 131 79 86 10.1016/S0166-4328(01)00349-7 11844574

[B69] Wilms H Rosenstiel P Unger T Deuschl G Lucius R Neuroprotection with angiotensin receptor antagonists: a review of the evidence and potential mechanisms Am J Cardiovasc Drugs 2005 5 245 253 10.2165/00129784-200505040-00004 15984907

[B70] Chrysant SG Chrysant GS The pleiotropic effects of Angiotensin receptor blockers J Clin Hypertens (Greenwich) 2006 8 261 268 10.1111/j.1524-6175.2005.05264.x 16596029 PMC8109722

[B71] Sonoda M Aoyagi T Takenaka K Uno K Nagai R A one-year study of the antiatherosclerotic effect of the angiotensin-II receptor blocker losartan in hypertensive patients. A comparison with angiotension-converting enzyme inhibitors Int Heart J 2008 49 95 103 10.1536/ihj.49.95 18360068

[B72] Royall DR Cordes JA Polk M CLOX: an executive clock drawing task J Neurol Neurosurg Psychiatry 1998 64 588 594 10.1136/jnnp.64.5.588 9598672 PMC2170069

[B73] Randolph C Tierney MC Mohr E Chase TN The Repeatable Battery for the Assessment of Neuropsychological Status (RBANS): preliminary clinical validity J Clin Exp Neuropsychol 1998 20 310 319 10.1076/jcen.20.3.310.823 9845158

[B74] Duff K Patton D Schoenberg MR Mold J Scott JG Adams RL Age- and education-corrected independent normative data for the RBANS in a community dwelling elderly sample Clin Neuropsychol 2003 17 351 366 14704885 10.1076/clin.17.3.351.18082

[B75] Bis JC Smith NL Psaty BM Heckbert SR Edwards KL Lemaitre RN Lumley T Rosendaal FR Angiotensinogen Met235Thr polymorphism, angiotensin-converting enzyme inhibitor therapy, and the risk of nonfatal stroke or myocardial infarction in hypertensive patients Am J Hypertens 2003 16 1011 1017 10.1016/j.amjhyper.2003.07.018 14643574

[B76] Washburn RA Ficker JL Physical Activity Scale for the Elderly (PASE): the relationship with activity measured by a portable accelerometer J Sports Med Phys Fitness 1999 39 336 340 10726435

[B77] Lawton MP Brody EM Assessment of older people: self-maintaining and instrumental activities of daily living Gerontologist 1969 9 179 186 5349366

[B78] Kurtz TW Griffin KA Bidani AK Davisson RL Hall JE Recommendations for blood pressure measurement in humans and experimental animals. Part 2: Blood pressure measurement in experimental animals: a statement for professionals from the subcommittee of professional and public education of the American Heart Association council on high blood pressure research Hypertension 2005 45 299 310 10.1161/01.HYP.0000150857.39919.cb 15611363

[B79] Pickering TG Hall JE Appel LJ Falkner BE Graves J Hill MN Jones DW Kurtz T Sheps SG Roccella EJ Recommendations for blood pressure measurement in humans and experimental animals: Part 1: blood pressure measurement in humans: a statement for professionals from the Subcommittee of Professional and Public Education of the American Heart Association Council on High Blood Pressure Research Hypertension 2005 45 142 161 15611362 10.1161/01.HYP.0000150859.47929.8e

[B80] Crum RM Anthony JC Bassett SS Folstein MF Population-based norms for the Mini-Mental State Examination by age and educational level Jama 1993 269 2386 2391 10.1001/jama.269.18.2386 8479064

[B81] Folstein MF Folstein SE McHugh PR "Mini-mental state". A practical method for grading the cognitive state of patients for the clinician J Psychiatr Res 1975 12 189 198 10.1016/0022-3956(75)90026-6 1202204

[B82] Shapiro AM Benedict RH Schretlen D Brandt J Construct and concurrent validity of the Hopkins Verbal Learning Test-revised Clin Neuropsychol 1999 13 348 358 10726605 10.1076/clin.13.3.348.1749

[B83] Kreiner DS Ryan JJ Memory and motor skill components of the WAIS-III Digit Symbol-Coding subtest Clin Neuropsychol 2001 15 109 113 11778571 10.1076/clin.15.1.109.1906

[B84] Wechsler D WMS-R: Wechsler Memory Scale--Revised: manual 1987 San Antonio: Psychological Corp: Harcourt Brace Jovanovich

[B85] Lipsitz LA Mukai S Hamner J Gagnon M Babikian V Dynamic regulation of middle cerebral artery blood flow velocity in aging and hypertension Stroke 2000 31 1897 1903 10926954 10.1161/01.str.31.8.1897

[B86] Newell DW Aaslid R Transcranial Doppler: clinical and experimental uses Cerebrovasc Brain Metab Rev 1992 4 122 143 1627439

[B87] Aaslid R Markwalder TM Nornes H Noninvasive transcranial Doppler ultrasound recording of flow velocity in basal cerebral arteries J Neurosurg 1982 57 769 774 10.3171/jns.1982.57.6.0769 7143059

[B88] Lipsitz LA Mukai S Hamner J Gagnon M Babikian V Dynamic Regulation of Middle Cerebral Artery Blood Flow Velocity in Aging and Hypertension Stroke 2000 31 1897 1903 10926954 10.1161/01.str.31.8.1897

[B89] Cartledge S Lawson N Aldosterone and renin measurements Ann Clin Biochem 2000 37 Pt 3 262 278 10.1258/0004563001899401 10817239

[B90] Underwood RH Williams GH The simultaneous measurement of aldosterone, cortisol, and corticosterone in human peripheral plasma by displacement analysis J Lab Clin Med 1972 79 848 862 5062996

[B91] Emanuel RL Cain JP Williams GH Double antibody radioimmunoassay of renin activity and angiotensin II in human peripheral plasma J Lab Clin Med 1973 81 632 640 4348744

[B92] Dicpinigaitis PV Angiotensin-Converting Enzyme Inhibitor-Induced Cough: ACCP Evidence-Based Clinical Practice Guidelines Chest 2006 129 169S 173 10.1378/chest.129.1_suppl.169S 16428706

[B93] Kostis JB Kim HJ Rusnak J Casale T Kaplan A Corren J Levy E Incidence and Characteristics of Angioedema Associated With Enalapril Arch Intern Med 2005 165 1637 1642 10.1001/archinte.165.14.1637 16043683

[B94] Diggle P Liang K Zeger S Analysis of Longitudinal Data 1994 Oxford University Press

[B95] McCullagh P Nelder J Generalized Liner Models 1987 London.: Chapman and Hall

[B96] Nilsson SE Read S Berg S Johansson B Melander A Lindblad U Low systolic blood pressure is associated with impaired cognitive function in the oldest old: longitudinal observations in a population-based sample 80 years and older Aging Clin Exp Res 2007 19 41 47 17332720 10.1007/BF03325209

[B97] Kilander L Nyman H Boberg M Lithell H The association between low diastolic blood pressure in middle age and cognitive function in old age. A population-based study Age Ageing 2000 29 243 248 10.1093/ageing/29.3.243 10855907

[B98] Axelsson J Reinprecht F Siennicki-Lantz A Elmstahl S Low ambulatory blood pressure is associated with lower cognitive function in healthy elderly men Blood Press Monit 2008 13 269 275 10.1097/MBP.0b013e32830d4be6 18799952

